# Trends in pathogens and antimicrobial resistance in culture-proven neonatal sepsis over a decade (2015–2024): the impact of COVID-19 pandemic

**DOI:** 10.3389/fcimb.2026.1913607

**Published:** 2026-09-03

**Authors:** Fatao Lin, Yushu Ma, Xiaojuan Li, Wenbo Yan, Hui Fu, Tiewei Li, Zhipeng Jin

**Affiliations:** 1 Department of Neonatology, Children’s Hospital Affiliated to Zhengzhou University, Henan Children’s Hospital, Zhengzhou Children’s Hospital, Zhengzhou, China; 2 School of Basic Medical Sciences, Zhengzhou University, Zhengzhou, China; 3 Department of Clinical Laboratory, Zhengzhou Key Laboratory of Children’s Infection and Immunity, Children’s Hospital Affiliated to Zhengzhou University, Henan Children’s Hospital, Zhengzhou Children’s Hospital, Zhengzhou, China; 4 Medical Intensive Care Unit, Children’s Hospital Affiliated to Zhengzhou University, Henan Children’s Hospital, Zhengzhou Children’s Hospital, Zhengzhou, China

**Keywords:** antimicrobial resistance, COVID-19, epidemiology, neonate, pathogens, sepsis

## Abstract

**Background:**

Neonatal sepsis remains a leading cause of mortality and long-term disability worldwide. The coronavirus disease 2019 (COVID-19) prevention measures effectively curbed viral transmission. However, evidence of their long-term impact on the pathogen spectrum and antimicrobial resistance in neonatal sepsis is limited.

**Methods:**

In this single-center retrospective observational study at Henan Children’s Hospital, 894 neonates with culture-confirmed sepsis (2015–2024) were enrolled. Using pandemic policy time points, we divided the study into three phases and compared pathogen spectrum and antimicrobial resistance.

**Results:**

Among neonates with sepsis, preterm proportion increased from 32.5% to 46.2%. The pathogen spectrum shifted from Gram-positive to Gram-negative predominance. *Coagulase-negative staphylococci* (CoNS) detection declined from 36.6% to 9.8%, a trend consistent across preterm/full-term and early-onset/late-onset subgroups. *Klebsiella pneumoniae (K. pneumoniae*) maintained high resistance to third-generation cephalosporins, while *Escherichia coli* (*E. coli*) showed a numerical increase in ceftazidime resistance. In contrast, CoNS and *Enterococcus faecium* (*E. faecium*) remained susceptible to vancomycin and linezolid. *E. coli* maintained favorable susceptibility to carbapenems and piperacillin-tazobactam, whereas *K. pneumoniae* showed a fluctuating carbapenem resistance trend that initially increased then declined, though it remained susceptible to tigecycline. All-cause mortality increased from 1.4% to 5.4%, with Gram-negative bacteria predominating in fatal cases.

**Conclusion:**

Enhanced hand hygiene and contact isolation are consistent with a sustained decline in CoNS, but show limited association with changes in Gram-negative bacilli. Routine surveillance and optimized empirical therapy remain crucial for improving neonatal sepsis outcomes.

## Introduction

Neonatal sepsis is defined as the invasion and proliferation of pathogenic microorganisms into normally sterile sites of a newborn, such as the bloodstream or cerebrospinal fluid, subsequently triggering systemic inflammatory response syndrome (Strunk et al., 2024; Subspecialty Group of Neonatology tSoP et al., 2024). Severe cases may progress to multiple organ dysfunction syndrome. This condition remains a leading cause of neonatal mortality and long-term disability worldwide (Fleischmann-Struzek et al., 2018). Globally, an estimated 5 million cases occur annually, with an incidence of 2,202 per 100,000 live births and a case fatality rate of 11%–19% (Fleischmann-Struzek et al., 2018). In addition, neonatal sepsis substantially increases the morbidity and mortality associated with other neonatal diseases, posing a major public health challenge worldwide (Strunk et al., 2024).

Neonatal sepsis exhibits substantial heterogeneity in pathogen spectrum and antimicrobial resistance across regions and populations, with continuous dynamic changes over time (Li et al., 2023). Henan Province, a highly populous region in central China, had 97.85 million permanent residents and 762,000 annual births in 2024, accounting for 8.0% of national births (Henan Provincial Bureau of Statistics NBoSSOiH, 2025; National Bureau of Statistics of China, 2025). Thus, the epidemiological characteristics of neonatal infections in this province hold significant reference value for public health decision-making in central China and nationwide. In late 2019, the COVID-19 pandemic posed an unprecedented challenge to global public health systems (Haldane et al., 2021; Wang and Naeem, 2025). Countries implemented a series of measures including enhanced hand hygiene, social distancing, and mask wearing to interrupt viral transmission (Talic et al., 2021). While these measures effectively contained the spread of severe acute respiratory syndrome coronavirus 2 (SARS-CoV-2), they also profoundly altered the epidemiological profiles of other infectious diseases (Zhang et al., 2023). Previous studies on pediatric respiratory pathogens showed that during the pandemic, the transmission intensity of common respiratory viruses and bacteria such as influenza virus, Mycoplasma pneumoniae, and respiratory syncytial virus decreased markedly, only to rebound to varying degrees after restrictions were relaxed (Li et al., 2023b; Li et al., 2023a; Li et al., 2023). However, unlike respiratory pathogens that spread mainly via droplets, neonatal sepsis is acquired through intrauterine, intrapartum, healthcare-associated, or community routes (Strunk et al., 2024). Whether the pandemic measures influenced the pathogen spectrum and antimicrobial resistance of neonatal sepsis, and whether such effects persisted after measures were lifted, remain unknown. A systematic report on the epidemiological changes of neonatal sepsis in Henan Province across the different COVID-19 prevention and control phases is still lacking.

This study conducted a systematic retrospective analysis of pathogen surveillance data from neonates with sepsis at Henan Children’s Hospital over a consecutive 10-year period from January 2015 to December 2024. The aim was to elucidate the pathogen composition, changing trends, and antimicrobial resistance profiles of neonatal sepsis across different phases of pandemic prevention and control, thereby providing evidence-based support for optimizing empirical anti-infective strategies in clinical practice.

## Materials and methods

### Study design and population

This was a single-center retrospective observational study conducted at Henan Children’s Hospital. The hospital is recognized as the National Children’s Regional Medical Center and the only tertiary grade-A specialized children’s hospital in Henan Province. Its Department of Neonatology, a National Key Clinical Specialty, admits over 3,000 neonates annually and maintains a well-established system for the diagnosis, treatment, and pathogen surveillance of neonatal infectious diseases. Neonates diagnosed with neonatal sepsis at this hospital between January 1, 2015, and December 31, 2024, were consecutively enrolled. Pathogen distribution and antimicrobial resistance trends were systematically analyzed over the 10-year period.

The diagnostic criteria for neonatal sepsis were based on the Chinese *Expert Consensus on Diagnosis and Management of Neonatal Bacteria Sepsis (2024) (*Subspecialty Group of Neonatology tSoP et al., 2024). Inclusion criteria were as follows: (1) Age of onset ≤ 28 days for full-term neonates, or ≤ 44 weeks of corrected gestational age for preterm neonates; (2) Presence of typical clinical manifestations related to infection; (3) Isolation of bacteria or fungi from single or multiple blood and/or cerebrospinal fluid cultures. For the common contaminant CoNS, cases were included in the analysis only if all of the following three conditions were met: (1) Presence of clinical manifestations related to infection; (2) At least two abnormalities among non-specific infection indicators, including white blood cell count, immature neutrophil proportion, platelet count, C-reactive protein, and procalcitonin; (3) Receipt of sensitive antimicrobial therapy for ≥ 5 days. Exclusion criteria were as follows: (1) Positive culture for CoNS that did not meet the above inclusion criteria or was clinically determined to be specimen contamination; (2) Incomplete clinical data; (3) Concurrent malignancy, congenital inborn errors of metabolism, autoimmune disease, or severe birth defects.

### Data collection

A standardized retrospective case report form was used to extract clinical data from the hospital information system (HIS), including age at onset, sex, gestational age at birth, birth weight, mode of delivery, blood and/or cerebrospinal fluid culture results, pathogen species, antimicrobial susceptibility test results, and clinical outcome (survival or in-hospital death). All variables were collected using uniform operational definitions.

Both blood and cerebrospinal fluid cultures were performed using the BD BACTEC FX automated system with an incubation period of 5 days. Duplicate isolates from the same patient were excluded from the analysis. Positive cultures were identified to the species level using the VITEK 2 automated identification system (bioMérieux, France) and the MALDI-TOF MS system (Bruker, Germany), with results verified by both platforms when necessary. Antimicrobial susceptibility testing was performed using VITEK 2 antimicrobial susceptibility cards (AST-GP67 for Gram-positive bacteria, AST-N335 for Gram-negative bacteria, AST-ST for streptococci, and AST-YS for fungi), supplemented by disc diffusion (Kirby-Bauer) and E-test methods when indicated.

Susceptibility results were interpreted according to the Clinical and Laboratory Standards Institute (CLSI) guidelines. The instruments used for culture, identification, and susceptibility testing remained unchanged throughout the 10-year study period. Although CLSI breakpoints were updated annually during this period, all retrospective susceptibility data were re-interpreted uniformly using the most current CLSI criteria applicable to each year’s results, rather than re-testing the stored isolates. This approach ensured consistency in susceptibility categorization across the entire study timeframe without introducing methodological variability from repeated testing. Quality control was performed using reference strains, including *E. coli* ATCC 25922, *Staphylococcus aureus* (*S. aureus)* ATCC 25923, *S. aureus* ATCC 29213, and *Pseudomonas aeruginosa (P. aeruginosa)* ATCC 27853.

### Operational definition

1. Time of onset.
  The date on which related clinical manifestations first appeared and the first blood and/or cerebrospinal fluid culture specimen was collected simultaneously.
2. Classification of cases.
  According to the age of onset, was classified into two categories.
  - Early-onset sepsis (EOS): age of onset ≤ 3 days.
  - Late-onset sepsis (LOS): age of onset > 3 days.
3. Classification of cases.
  According to gestational age at birth, cases were classified into two categories.
  - Preterm neonates: gestational age < 37 weeks.
  - Full-term neonates: gestational age ≥ 37 weeks.
4. Classification of study periods.
  According to the implementation and adjustment milestones of China’s COVID-19 prevention and control policies, the study period was divided into three phases.
  - Pre-pandemic period: January 2015 to December 2019.
  - Pandemic period: January 2020 to December 2022.
  - Post-pandemic period: January 2023 to December 2024.
5. In-hospital death.
  In-hospital death included any death occurring between admission and discharge, including death on the same day when the critical condition of neonates led parents to request cessation of treatment due to concerns about a poor prognosis.

### Statistical analysis

All statistical analyses were performed using SPSS version 27.0 (IBM SPSS Statistics) and R version 4.6.1 (R Foundation for Statistical Computing), and graphs were created using GraphPad Prism version 10.4 (GraphPad Software). For continuous variables, those following a normal distribution were expressed as mean ± standard deviation (x ± s), and comparisons among multiple groups were performed using one-way analysis of variance (ANOVA). For continuous variables not following a normal distribution, data were expressed as median (interquartile range) [M (P25, P75)], and comparisons among multiple groups were performed using the Kruskal-Wallis H test. Categorical variables were expressed as percentages (%), and comparisons among groups were performed using the chi-square (χ²) test or Fisher’s exact test. For post-hoc pairwise comparisons among multiple groups, we applied the Bonferroni correction. For analyses of pathogen spectrum composition and resistance trends across the three periods, we further applied the Benjamini-Hochberg procedure to adjust the false discovery rate (FDR). An FDR-adjusted P-value < 0.05 was considered statistically significant for the resistance-related multiple comparisons, and a two-sided P-value < 0.05 was used for all other analyses.

### Ethical approval

This study adhered to the ethical principles of the Declaration of Helsinki and was approved by the Medical Ethics Committee of Henan Children’s Hospital (2025-IIT-041-001). All personally identifiable information was anonymized, and only study-related variables were retained. Given the retrospective design, the requirement for informed consent was exempted by the Ethics Review Committee of Henan Children’s Hospital (2025-IIT-041-001).

## Results

### Admission trends, incidence, and baseline characteristics of the study population

**Figure 1:**
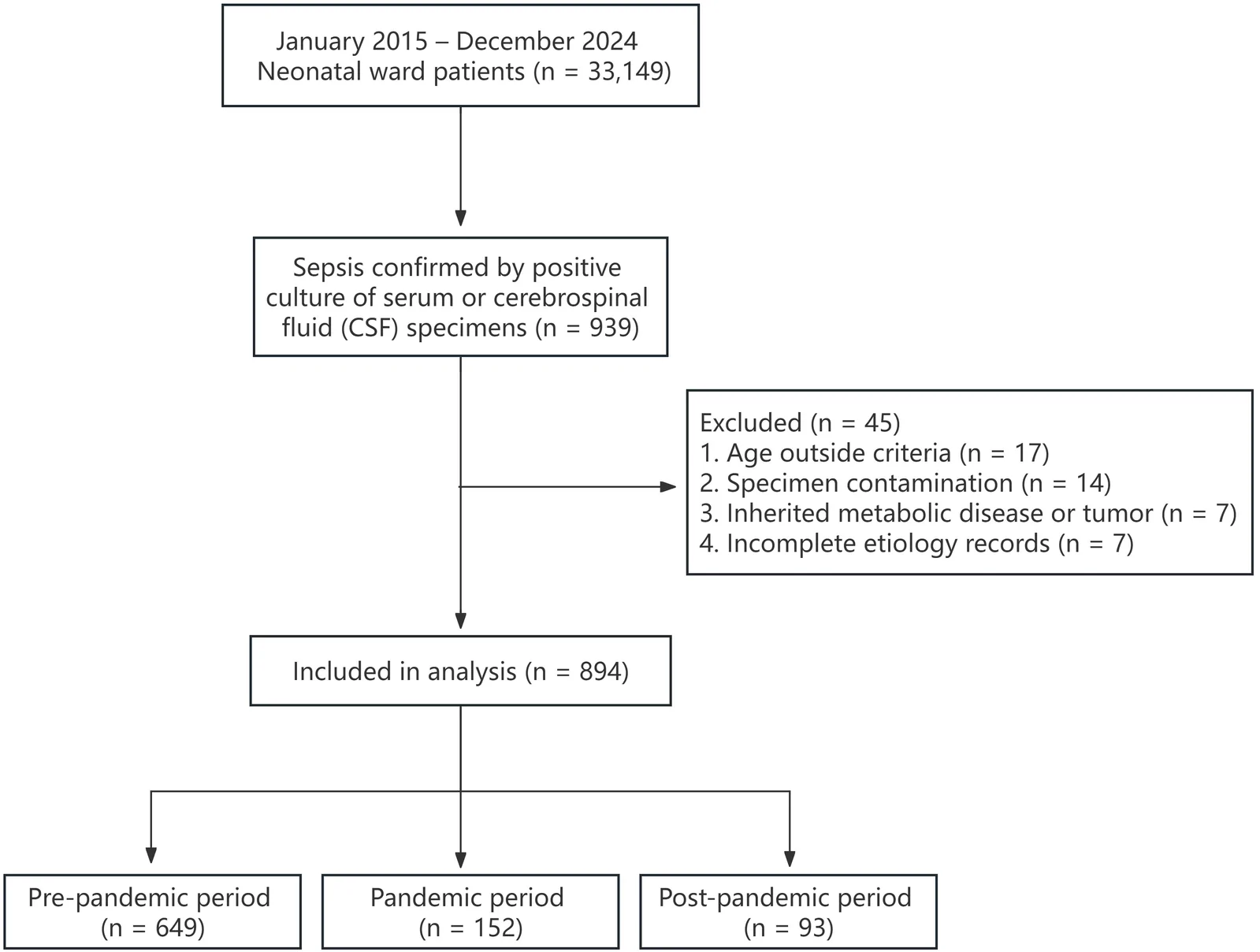
Flowchart of case screening for the enrolled study.

Between January 2015 and December 2024, a total of 33,149 neonates were admitted to the Department of Neonatology at Henan Children’s Hospital. Given that the three study periods differed in duration (5 years pre-pandemic, 3 years during the pandemic, and 2 years post-pandemic period), comparisons of hospital admission volumes across periods were performed using annualized data. The average annual number of admissions in the three periods was 4,134/year (pre-pandemic period), 2,473/year (pandemic period), and 2,530/year (post-pandemic period). During the same study period, 939 neonates with suspected sepsis were screened. After applying the inclusion and exclusion criteria, 894 neonates were confirmed as sepsis cases (**Figure 1**), yielding an overall incidence of 2.70%. The incidence rates in the three periods were 3.14%, 2.05%, and 1.84%, respectively, and the overall difference was statistically significant (P < 0.001). Pairwise comparisons showed that the pre-pandemic period rate was significantly higher than those in both the pandemic and post-pandemic periods (both P < 0.05), whereas no significant difference was observed between the latter two periods (**Supplementary Figure 1**). A year-by-year decomposition analysis of the post-pandemic period revealed no statistically significant differences between 2023 and 2024 in sepsis incidence, all-cause mortality rate, CoNS detection rate, or the proportion of preterm neonates (all P > 0.05; **Supplementary Table 2**).

Among the 894 confirmed neonatal sepsis cases, males and patients with LOS comprised the majority in their respective groups. Baseline characteristics are detailed in **Table 1**. No statistically significant differences were observed among the three periods in sex distribution, mode of delivery, sepsis type composition, or age at onset (all P > 0.05). The proportion of preterm neonates increased progressively from 32.51% in the pre-pandemic period to 38.82% during the pandemic and further to 46.24% in the post-pandemic period (P = 0.019). Concurrently, both median gestational age and median birth weight exhibited declining trends (both P < 0.001). The in-hospital mortality rate rose from 1.39% to 3.29% and then to 5.38% (P = 0.023).

**Table 1:** Baseline characteristics of neonates with sepsis.

Characteristic	Total (N = 894)	Pre-pandemic (n = 649)	Pandemic (n = 152)	Post-pandemic (n = 93)	*P*
Sex, n (%)					0.365
Male	555 (62.08)	412 (63.48)	88 (57.89)	55 (59.14)	
Female	339 (37.92)	237 (36.52)	64 (42.11)	38 (40.86)	
Mode of delivery, n (%)					0.254
Vaginal delivery	452 (50.56)	339 (52.23)	69 (45.39)	44 (47.31)	
Cesarean section	442 (49.44)	310 (47.77)	83 (54.61)	49 (52.69)	
type, n (%)					0.984
EOS	143 (16.00)	103 (15.87)	25 (16.45)	15 (16.13)	
LOS	751 (84.00)	546 (84.13)	127 (83.55)	78 (83.87)	
Age of onset (days)	13.00 (7.00, 21.00)	13.00 (6.00, 21.00)	13.00 (7.00, 22.00)	11.00 (5.00, 19.00)	0.242
Gestational age (weeks)	38.14 (34.14, 39.57)	38.43 (35.00, 39.71)	37.57 (31.93, 39.43)^a^	37.43 (31.71, 38.86)^a^	<0.001
Gestational age, n (%)					0.019
Preterm	313 (35.01)	211 (32.51)	59 (38.82)	43 (46.24)^a^	
Full-term	581 (64.99)	438 (67.49)	93 (61.18)	50 (53.76)	
Birth weight (kg)	3.00 (2.09, 3.50)	3.10 (2.23, 3.50)	2.83 (1.66, 3.40)^a^	2.74 (1.70, 3.31)^a^	<0.001
Birth weight, n (%)					0.002
< 1.5 kg	117 (13.09)	68 (10.48)	30 (19.74)^a^	19 (20.43)^a^	
1.5-2.5 kg	166 (18.57)	118 (18.18)	26 (17.11)	22 (23.66)	
≥ 2.5 kg	611 (68.34)	463 (71.34)	96 (63.16)	52 (55.91)^a^	

^a^Compared with the first period, P<0.05.

EOS, Early-onset sepsis; LOS, Late-onset sepsis.

### Pathogen distribution and epidemiological trends

Among the 894 neonatal sepsis cases, a total of 946 pathogenic bacteria were isolated. The overall composition of Gram-positive bacteria, Gram-negative bacteria, and fungi across the three periods is presented in **Table 2**. After FDR adjustment, shifts in the pathogen spectrum were observed: the overall proportion of Gram-positive bacteria declined, whereas that of Gram-negative bacteria increased (FDR-adjusted P = 0.002). In the pre-pandemic period, Gram-positive bacteria predominated (53.93%), whereas during the pandemic and post-pandemic periods, Gram-negative bacteria became the dominant group, accounting for 50.30% and 59.80%, respectively. No significant difference was observed in the overall proportion of fungi among the three periods (FDR-adjusted P = 0.379). Among specific species, the proportion of CoNS decreased from 36.59% in the pre-pandemic period to 9.80% in the post-pandemic period (FDR-adjusted P = 0.005), with the post-pandemic proportion significantly lower than both the pre-pandemic and pandemic periods (Bonferroni-adjusted P < 0.05 for both). The proportions of *E. coli* and *P. aeruginosa* both exhibited increasing trends (FDR-adjusted P = 0.005 for both). The detection rates of *Listeria monocytogenes* (*L. monocytogenes*), *Stenotrophomonas maltophilia* (*S. maltophilia*), as well as the Gram-negative bacteria and fungal species that were collectively categorized as “Others” in **Table 2** differed significantly across the three periods (all FDR-adjusted P < 0.05). In contrast, no significant differences were observed for the remaining pathogens (all FDR-adjusted P > 0.05; **Table 2**).

**Table 2:** Differences in pathogen distribution across the three study periods.

Pathogen	Total (N = 946)	Pre-pandemic (n = 675)	Pandemic (n = 169)	Post-pandemic (n = 102)	*P*	*P^#^*
Gram-positive bacteria, n (%)	470 (49.68)	364 (53.93)	75 (44.38)	31 (30.39) ^a^	<0.001	0.002
*CoNS*	297 (31.40)	247 (36.59)	40 (23.67) ^ab^	10 (9.80) ^a^	<0.001	0.005
*E. faecium*	44 (4.65)	27 (4.00)	12 (7.10)	5 (4.90)	0.207	0.331
*S. aureus*	41 (4.33)	32 (4.74)	5 (2.96)	4 (3.92)	0.663	0.816
*L. monocytogenes*	20 (2.11)	9 (1.33)	5 (2.96)	6 (5.88) ^a^	0.010	0.027
*GBS*	19 (2.01)	15 (2.22)	3 (1.78)	1 (0.98)	0.869	0.891
Others	49 (5.18)	34 (5.04)	10 (5.92)	5 (4.90)	0.891	0.891
Gram-negative bacteria, n (%)	409 (43.23)	263 (38.96)	85 (50.30) ^a^	61 (59.80) ^a^	<0.001	0.002
*K. pneumoniae*	162 (17.12)	111 (16.44)	34 (20.12)	17 (16.67)	0.521	0.695
*E. coli*	126 (13.32)	71 (10.52)	31 (18.34) ^a^	24 (23.53) ^a^	<0.001	0.005
*S. maltophilia*	31 (3.28)	29 (4.30)	1 (0.59)	1 (0.98)	0.018	0.041
*E. cloacae*	16 (1.69)	14 (2.07)	2 (1.18)	0 (0.00)	0.391	0.569
*P. aeruginosa*	10 (1.06)	2 (0.30)	2 (1.18)	6 (5.88) ^a^	<0.001	0.005
Others	64 (6.77)	36 (5.33)	15 (8.88)	13 (12.75) ^a^	0.010	0.027
Fungi, n (%)	67 (7.08)	48 (7.11)	9 (5.33)	10 (9.80)	0.379	0.379
*C. albicans*	29 (3.07)	23 (3.41)	1 (0.59)	5 (4.90)	0.054	0.108
*C. parapsilosis*	15 (1.59)	14 (2.07)	0 (0.00)	1 (0.98)	0.126	0.224
*C. glabrata*	7 (0.74)	5 (0.74)	1 (0.59)	1 (0.98)	0.835	0.891
Others	16 (1.69)	6 (0.89)	7 (4.14) ^a^	3 (2.94)	0.006	0.024

(1) P values were derived from the chi-square test comparing resistance rates across the three periods. A P value of < 0.05 was considered statistically significant.

(2) ^a^ compared with the first period; ^b^ compared with the third period, P<0.05.

(3) P^#^ values were adjusted for multiple comparisons using the FDR procedure, applied separately to the pathogen category and to each specific bacterial species group. An FDR-adjusted P^#^ < 0.05 was considered statistically significant.

CoNS, *coagulase-negative staphylococci*; *E. faecium*, *Enterococcus faecium*; *S. aureus*, *Staphylococcus aureus*; *L. monocytogenes*, *Listeria monocytogenes*; *GBS*, *Group B Streptococcus*; *K. pneumoniae*, *Klebsiella pneumoniae*; *E. coli*, *Escherichia coli*; *S. maltophilia*, *Stenotrophomonas maltophilia*; *E. cloacae*, *Enterobacter cloacae*; *P. aeruginosa*, *Pseudomonas aeruginosa*; *C. albicans*, *Candida albicans*; *C. parapsilosis*, *Candida parapsilosis*; *C. glabrata*, *Candida glabrata.*

Among the 894 confirmed cases, 804 (89.93%) were positive by blood culture alone, 54 (6.04%) by cerebrospinal fluid (CSF) culture alone, and 36 (4.03%) by both blood and CSF cultures (**Supplementary Figure 2A**). Annual trend analysis revealed that the detection rate of Gram-positive bacteria gradually decreased from 61.70% in 2015 to 28.33% in 2024, whereas the detection rate of Gram-negative bacteria increased from 34.04% to 61.67% (with a transient decline to 42.31% in 2021). The detection rate of fungi fluctuated between 0% and 11.54% across the years (**Supplementary Figure 2B**).

### Pathogen distribution stratified by gestational age

Among preterm neonates, Gram-negative bacteria predominated across all three periods, with *K. pneumoniae* consistently exhibiting the highest detection rate. No statistically significant differences were observed in the overall pathogen distribution among the three groups (all FDR-adjusted P > 0.05). In contrast, among full-term neonates, significant differences in the overall distribution of Gram-positive and Gram-negative bacteria were observed across the three periods (FDR-adjusted P = 0.002 for both). *E. coli* showed the highest detection rate in the post-pandemic period (40.74%, FDR-adjusted P = 0.008), while the detection rate of CoNS exhibited a declining trend (FDR-adjusted P = 0.008) (**Table 3**).

**Table 3:** Differences in pathogen distribution between preterm and full-term neonates with neonatal sepsis.

Pathogen	Preterm neonates						Full-term neonates					
	Total (n=334)	Pre-pandemic (n = 222)	Pandemic (n = 64)	Post-pandemic (n = 48)	*P*	*P^#^*	Total (n=612)	Pre-pandemic (n = 453)	Pandemic (n =105)	Post-pandemic (n =54)	*P*	*P^#^*
Gram-positive bacteria, n (%)	108 (32.34)	73 (32.88)	22 (34.38)	13 (27.08)	0.685	0.685	362 (59.15)	291 (64.24)	53 (50.48)^a^	18 (33.33)^a^	<0.001	0.002
*CoNS*	47 (14.07)	39 (17.57)	6 (9.38)	2 (4.17)	0.026	0.132	252 (41.18)	209 (46.14)	35 (33.33)^b^	8 (14.81)^a^	<0.001	0.008
*E. faecium*	27 (8.08)	15 (6.76)	9 (14.06)	3 (6.25)	0.155	0.310	17 (2.78)	12 (2.65)	3 (2.86)	2 (3.70)	0.834	1.000
*S. aureus*	4 (1.20)	2 (0.90)	1 (1.56)	1 (2.08)	0.411	0.598	37 (6.05)	30 (6.62)	4 (3.81)	3 (5.56)	0.620	1.000
*L. monocytogenes*	11 (3.29)	5 (2.25)	3 (4.69)	3 (6.25)	0.210	0.373	9 (1.47)	4 (0.88)	2 (1.90)	3 (5.56)^a^	0.028	0.090
*GBS*	2 (0.60)	2 (0.90)	0 (0.00)	0 (0.00)	1.000	1.000	17 (2.78)	13 (2.87)	3 (2.86)	1 (1.85)	1.000	1.000
Others	17 (5.09)	10 (4.50)	3 (4.69)	4 (8.33)	0.523	0.644	30 (4.90)	23 (5.08)	6 (5.71)	1 (1.85)	0.661	1.000
Gram-negative bacteria, n (%)	174 (52.10)	112 (50.45)	36 (56.25)	26 (54.17)	0.682	0.685	235 (38.40)	151 (33.33)	49 (46.67)^a^	35 (64.81)^a^	<0.001	0.002
*K. pneumoniae*	106 (31.74)	74 (33.33)	21 (32.81)	11 (22.92)	0.364	0.582	56 (9.15)	37 (8.17)	13 (12.38)	6 (11.11)	0.316	0.632
*E. coli*	21 (6.29)	10 (4.50)	9 (14.06)^a^	2 (4.17)	0.028	0.132	105 (17.16)	61 (13.47)	22 (20.95)^b^	22 (40.74)^a^	<0.001	0.008
*S. maltophilia*	10 (2.99)	8 (3.60)	1 (1.56)	1 (2.08)	0.888	0.947	21 (3.43)	21 (4.64)	0 (0.00)	0 (0.00)	0.018	0.072
*E. cloacae*	5 (1.50)	5 (2.25)	0 (0.00)	0 (0.00)	0.507	0.644	11 (1.80)	9 (1.99)	2 (1.90)	0 (0.00)	0.770	1.000
*P. aeruginosa*	4 (1.20)	0 (0.00)	1 (1.56)	3 (6.25)^a^	0.004	0.064	6 (0.98)	2 (0.44)	1 (0.95)	3 (5.56)^a^	0.010	0.053
Others	11 (3.29)	15 (6.76)	4 (6.25)	9 (18.75)^a^	0.033	0.132	36 (5.88)	21 (4.64)	11 (10.48)	4 (7.41)	0.054	0.123
Fungi, n (%)	52 (15.57)	37 (16.67)	6 (9.38)	9 (18.75)	0.295	0.685	15 (2.45)	11 (2.43)	3 (2.86)	1 (1.85)	0.903	0.903
*C. albicans*	23 (6.89)	17 (7.66)	1 (1.56)	5 (10.42)	0.119	0.272	6 (0.98)	6 (1.32)	0 (0.00)	0 (0.00)	0.771	1.000
*C. parapsilosis*	14 (4.19)	13 (5.86)	0 (0.00)	1 (2.08)	0.082	0.262	1 (0.16)	1 (0.22)	0 (0.00)	0 (0.00)	1.000	1.000
*C. glabrata*	5 (1.50)	3 (1.35)	1 (1.56)	1 (2.08)	0.812	0.928	2 (0.33)	2 (0.44)	0 (0.00)	0 (0.00)	1.000	1.000
Others	10 (2.99)	4 (1.80)	4 (6.25)	2 (4.17)	0.104	0.272	6 (0.98)	2 (0.44)	3 (2.86)	1 (1.85)	0.048	0.123

(1) P values were derived from the chi-square test comparing resistance rates across the three periods. A P value of < 0.05 was considered statistically significant.

(2)^a^ compared with the first period;^b^ compared with the third period, P<0.05.

(3) P^#^ values were adjusted for multiple comparisons using the FDR procedure, applied separately to the pathogen category and to each specific bacterial species group. An FDR-adjusted P^#^ < 0.05 was considered statistically significant.

CoNS, *coagulase-negative staphylococci*; *E. faecium*, *Enterococcus faecium*; *S. aureus*, *Staphylococcus aureus*; *L. monocytogenes*, *Listeria monocytogenes*; *GBS*, *Group B Streptococcus*; *K. pneumoniae*, *Klebsiella pneumoniae*; *E. coli*, *Escherichia coli*; *S. maltophilia*, *Stenotrophomonas maltophilia*; *E. cloacae*, *Enterobacter cloacae*; *P. aeruginosa*, *Pseudomonas aeruginosa*; *C. albicans*, *Candida albicans*; *C. parapsilosis*, *Candida parapsilosis*; *C. glabrata*, *Candida glabrata.*

### Pathogen distribution stratified by age of onset

Among EOS cases, Gram-positive bacteria predominated throughout the three periods. No significant differences were observed in the overall composition of Gram-positive bacteria, Gram-negative bacteria, or fungi among the three groups, nor in the detection rates of individual bacterial species (all FDR-adjusted P > 0.05). In contrast, among LOS cases, the overall proportion of Gram-negative bacteria increased over time, whereas that of Gram-positive bacteria decreased (all FDR-adjusted P < 0.01). The detection rate of CoNS declined progressively, while that of *E. coli* rose (all FDR-adjusted P < 0.01). In addition, period-specific differences in the detection rates of *P. aeruginosa*, and the Gram-negative and fungal listed as “Others” were also statistically significant (FDR-adjusted P = 0.019 for all) (**Table 4**).

**Table 4:** Differences in pathogen distribution between EOS and LOS neonatal sepsis.

Pathogen	EOS						LOS					
	Total (n=153)	Pre-pandemic (n = 108)	Pandemic (n = 26)	Post-pandemic (n = 19)	*P*	*P^#^*	Total (n=793)	Pre-pandemic (n = 567)	Pandemic (n =143)	Post-pandemic (n = 83)	*P*	*P^#^*
Gram-positive bacteria, n (%)	101 (66.01)	74 (68.52)	17 (65.38)	10 (52.63)	0.402	0.402	369 (46.53)	290 (51.15)	58 (40.56)	21 (25.30)^a^	<0.001	0.002
*CoNS*	59 (38.56)	48 (44.44)	9 (34.62)	2 (10.53)^a^	0.018	0.108	240 (30.26)	200 (35.27)^b^	32 (22.38)^ab^	8 (9.64)^a^	<0.001	0.008
*E. faecium*	3 (1.96)	3 (2.78)	0 (0.00)	0 (0.00)	1.000	1.000	41 (5.17)	24 (4.23)	12 (8.39)	5 (6.02)	0.103	0.210
*S. aureus*	2 (1.31)	2 (1.85)	0 (0.00)	0 (0.00)	1.000	1.000	39 (4.92)	30 (5.29)	5 (3.50)	4 (4.82)	0.745	0.795
*L. monocytogenes*	19 (12.42)	9 (8.33)	5 (19.23)	5 (26.32)	0.035	0.140	1 (0.13)	0 (0.00)	0 (0.00)	1 (1.20)	0.105	0.210
*GBS*	3 (1.96)	2 (1.85)	0 (0.00)	1 (5.26)	0.395	0.663	16 (2.02)	13 (2.29)	3 (2.10)	0 (0.00)	0.550	0.677
Others	15 (9.80)	10 (9.26)	3 (11.54)	2 (10.53)	0.836	1.000	32 (4.04)	23 (4.06)	6 (4.20)	3 (3.61)	1.000	1.000
Gram-negative bacteria, n (%)	51 (33.33)	34 (31.48)	8 (30.77)	9 (47.37)	0.381	0.402	358 (45.15)	229 (40.39)	77 (53.85)^a^	52 (62.65)^a^	<0.001	0.002
*K. pneumoniae*	9 (5.88)	4 (3.70)	3 (11.54)	2 (10.53)	0.125	0.375	153 (19.29)	107 (18.87)	31 (21.68)	15 (18.07)	0.717	0.795
*E. coli*	16 (10.46)	12 (11.11)	1 (3.85)	3 (15.79)	0.442	0.663	110 (13.87)	59 (10.41)	30 (20.98)^a^	21 (25.30)^a^	<0.001	0.008
*S. maltophilia*	9 (5.88)	9 (8.33)	0 (0.00)	0 (0.00)	0.192	0.461	22 (2.77)	20 (3.53)	1 (0.70)	1 (1.20)	0.150	0.240
*E. cloacae*	0 (0.00)	0 (0.00)	0 (0.00)	0 (0.00)	/	/	16 (2.02)	14 (2.47)	2 (1.40)	0 (0.00)	0.389	0.519
*P. aeruginosa*	3 (1.96)	0 (0.00)	1 (3.85)	2 (10.53)^a^	0.014	0.108	7 (0.88)	2 (0.35)	1 (0.70)	4 (4.82)^a^	0.004	0.019
Others	14 (9.15)	9 (8.33)	3 (11.54)	2 (10.53)	0.741	0.988	50 (6.31)	27 (4.76)	12 (8.39)	11 (13.25)^a^	0.006	0.019
Fungi, n (%)	1 (0.65)	0 (0.00)	1 (3.85)	0 (0.00)	0.294	0.402	66 (8.32)	48 (8.47)	8 (5.59)	10 (12.05)	0.232	0.232
*C. albicans*	0 (0.00)	0 (0.00)	0 (0.00)	0 (0.00)	/	/	29 (3.66)	23 (4.06)	1 (0.70)^b^	5 (6.02)	0.045	0.120
*C. parapsilosis*	0 (0.00)	0 (0.00)	0 (0.00)	0 (0.00)	/	/	15 (1.89)	14 (2.47)	0 (0.00%)	1 (1.20)	0.121	0.215
*C. glabrata*	1 (0.65)	0 (0.00)	1 (3.85)	0 (0.00)	0.294	0.588	6 (0.76)	5 (0.88)	0 (0.00)	1 (1.20)	0.388	0.519
Others	0 (0.00)	0 (0.00)	0 (0.00)	0 (0.00)	/	/	16 (2.02)	6 (1.06)	7 (4.90)^a^	3 (3.61)	0.006	0.019

(1) P values were derived from the chi-square test comparing resistance rates across the three periods. A P value of < 0.05 was considered statistically significant.

(2)^a^ compared with the first period;^b^ compared with the third period, P<0.05.

(3) P^#^ values were adjusted for multiple comparisons using the FDR procedure, applied separately to the pathogen category and to each specific bacterial species group. An FDR-adjusted P^#^ < 0.05 was considered statistically significant.

(4) “/” indicates that the antibiotic is not applicable to the corresponding pathogen or that susceptibility testing was not performed.

CoNS, *coagulase-negative staphylococci*; *E. faecium*, *Enterococcus faecium*; *S. aureus*, *Staphylococcus aureus*; *L. monocytogenes*, *Listeria monocytogenes*; *GBS*, *Group B Streptococcus*; *K. pneumoniae*, *Klebsiella pneumoniae*; *E. coli*, *Escherichia coli*; *S. maltophilia*, *Stenotrophomonas maltophilia*; *E. cloacae*, *Enterobacter cloacae*; *P. aeruginosa*, *Pseudomonas aeruginosa*; *C. albicans*, *Candida albicans*; *C. parapsilosis*, *Candida parapsilosis*; *C. glabrata*, *Candida glabrata;* EOS, Early-onset sepsis; LOS, Late-onset sepsis.

### Antimicrobial resistance trends of major pathogens

Among Gram-positive bacteria, CoNS and *E. faecium* were the predominant species. Throughout the entire study period, the resistance rates of CoNS to vancomycin and linezolid remained consistently below 3.00%, whereas those to penicillin G and erythromycin were persistently above 70.00% (**Table 5**). After FDR adjustment, no statistically significant differences in resistance rates were observed across the three periods for CoNS. *E. faecium* maintained 100.00% susceptibility to linezolid, vancomycin, and tigecycline throughout all three periods, while its resistance rates to penicillin G and erythromycin ranged between 70.00% and 100.00%, and the resistance rate to ciprofloxacin showed a decreasing trend from 88.46% to 25.00%.

**Table 5:** Antimicrobial susceptibility of CoNS and *E. faecium* isolated in different periods.

Antibiotics	CoNS					*E. faecium*				
	Pre-pandemic n, (%)	Pandemic n, (%)	Post-pandemic n, (%)	*P*	*P^#^*	Pre-pandemic n, (%)	Pandemic n, (%)	Post-pandemic n, (%)	*P*	*P^#^*
Oxacillin	186 (75.92)	32 (84.21)	8 (80.00)	0.522	0.979	/	/	/	/	/
Cotrimoxazole	126 (54.31)	13 (34.21)	5 (50.00)	0.068	0.340	/	/	/	/	/
Erythromycin	214 (86.99)	27 (72.97)	8 (80.00)	0.067	0.340	25 (92.59)	7 (70.00)	4 (100.00)*	0.148	0.148
Ciprofloxacin	66 (29.46)	10 (34.48)	4 (40.00)	0.656	0.989	23 (88.46)	4 (57.14)*	1 (25.00)^a^ *	0.010	0.010
Clindamycin	173 (70.04)	18 (48.65)^a^	4 (40.00)	0.006	0.090	26 (96.30)	9 (100.00)*	4 (100.00)*	1.000	1.000
Rifampicin	37 (15.16)	6 (15.79)	2 (20.00)	0.819	1.000	/	/	/	/	/
Linezolid	3 (1.24)	0 (0.00)	0 (0.00)	1.000	1.000	0 (0.00)	0 (0.00)	0 (0.00)*	/	/
Moxifloxacin	3 (23.08)	4 (12.90)	3 (30.00)	0.397	0.909	1 (100.00)*	7 (87.50)*	4 (100.00)*	1.000	1.000
Penicillin G	1 (100.00)*	12 (92.31)	10 (100.00)	1.000	1.000	/	7 (100.00)*	4 (100.00)*	/	/
Gentamicin	82 (33.20)	7 (18.42)	2 (20.00)	0.157	0.489	/	/	/	/	/
Tetracycline	59 (25.00)	6 (18.18)	2 (20.00)	0.775	1.000	19 (76.00)	5 (55.56)*	2 (50.00)*	0.378	0.378
Tigecycline	0 (0.00)*	0 (0.00)	0 (0.00)	/	/	0 (0.00)*	0 (0.00)*	0 (0.00)*	/	/
Vancomycin	0 (0.00)	1 (2.63)	0 (0.00)	0.163	0.489	0 (0.00)	0 (0.00)	0 (0.00)*	/	/
Levofloxacin	3 (30.00)	4 (30.77)	5 (50.00)	0.659	0.989	1 (50.00)*	6 (66.67)*	1 (25.00)*	0.491	0.491
Quinupristin	3 (1.83)	1 (7.14)	0 (0.00)	0.424	0.909	1 (4.76)	0 (0.00)*	1 (25.00)*	0.276	0.276
Ampicillin	25 (96.15)	10 (100.00)	4 (100.00)*	1.000	1.000	/	/	/	/	/
High-Level Streptomycin	0 (0.00)*	2 (40.00)*	0 (0.00)*	0.556	/	/	/	/	/	/
High-Level Gentamicin	27 (100.00)	5 (71.43)^a^ *	4 (100.00)*	0.038	/	/	/	/	/	/

(1) Data are presented as number of resistant isolates/total number of isolates tested (resistance rate %). Denominators vary because some isolates were not tested for the corresponding antimicrobial agents.

(2) P values were derived from the chi-square test comparing resistance rates across the three periods. A P value of < 0.05 was considered statistically significant.

(3) P^#^ values were adjusted for multiple comparisons using the Benjamini-Hochberg false discovery rate (FDR) procedure, applied separately to each antibiotic-pathogen combination group. An FDR-adjusted P^#^ < 0.05 was considered statistically significant.

(4) “/” indicates that the antibiotic is not applicable to the corresponding pathogen or that susceptibility testing was not performed.

(5) *: The number of tested isolates in this cell is < 10, and percentages should be interpreted with caution.

CoNS, *coagulase-negative staphylococci*; *E. faecium*, *Enterococcus faecium.*

Among Gram-negative bacteria, *K. pneumoniae* and *E. coli* were the major pathogens. *K. pneumoniae* maintained 100.00% susceptibility to tigecycline, colistin, and polymyxin B across all three periods. However, its resistance rates to penicillins (e.g., ampicillin, piperacillin), β-lactamase inhibitor combinations (e.g., amoxicillin-clavulanate, piperacillin-tazobactam), and third-generation cephalosporins (e.g., cefotaxime, ceftazidime) remained at relatively high levels, while the resistance rate to carbapenems (e.g., imipenem, meropenem) increased during the pandemic period and subsequently declined in the post-pandemic period (**Table 6**). In contrast, *E. coli* exhibited relatively high resistance rates to penicillins and third-generation cephalosporins, but showed lower resistance rates to amikacin, carbapenems, β-lactamase inhibitor combinations, and tigecycline. After FDR adjustment, no statistically significant differences in resistance rates were observed for either bacterium across the three periods (all FDR-adjusted P > 0.05). Detailed susceptibility results for other antimicrobial agents are provided in **Supplementary Table 1**.

**Table 6:** Antimicrobial susceptibility of *K. pneumoniae* and *E. coli* isolated in different periods.

Antibiotics	*K. pneumoniae*					*E. coli*				
	Pre-pandemic n, (%)	Pandemic n, (%)	Post-pandemic n, (%)	*P*	*P^#^*	Pre-pandemic n, (%)	Pandemic n, (%)	Post-pandemic n, (%)	*P*	*P^#^*
Amikacin	43 (39.81)	13 (46.43)^b^	1 (6.25)^a^	0.020	0.148	0 (0.00)	0 (0.00)	0 (0.00)	/	/
Amoxicillin-Clavulanate	72 (70.59)	26 (86.67)^b^	8 (53.33)	0.049	0.148	5 (8.06)	6 (19.35)	3 (13.64)	0.270	0.714
Ampicillin	100 (100.00)	4 (100.00)*	11 (91.67)	0.138	0.191	51 (86.44)	11 (100.00)	12 (85.71)	0.576	0.816
Doripenem	3(60.0%)*	18(75.0%)	6(42.9%)	0.14	0.191	0 (0.00)*	1 (4.55)	0 (0.00)	1.000	1.000
Meropenem	60(60.0%)	21(75.0%)	7(43.8%)	0.11	0.183	1 (1.59)	1 (3.23)	0 (0.00)	1.000	1.000
Norfloxacin	4 (40.00)	24 (80.00)	2 (100.00)*	0.054	0.148	8 (80.00)	13 (48.15)	5 (50.00)	0.287	0.714
Piperacillin	50 (87.72)	23 (95.83)	14 (100.00)	0.366	0.422	31 (81.58)	21 (95.45)	20 (90.91)	0.314	0.714
Piperacillin-Tazobactam	67 (65.05)	26 (86.67)	11 (73.33)	0.063	0.148	1 (1.49)	3 (9.68)	3 (13.64)	0.033	0.281
Tigecycline	0 (0.00)	0 (0.00)	0 (0.00)	/	/	0 (0.00)*	0 (0.00)	0 (0.00)	/	/
Ticarcillin	5 (100.00)*	24 (100.00)	15 (100.00)	/	/	3 (75.00)*	21 (95.45)	20 (90.91)	0.378	0.714
Ticarcillin-Clavulanate	4 (100.00)*	15 (100.00)^b^	10 (62.50)	0.019	0.148	0 (0.00)*	1 (5.26)	3 (13.64)	0.680	0.889
Cefoperazone	11 (100.00)	3 (100.00)*	2 (100.00)*	/	/	3 (33.33)*	0 (0.00)*	2 (40.00)*	1.000	/
Cefoperazone-Sulbactam	4 (50.00)*	23 (82.14)	9 (56.25)	0.069	0.148	0 (0.00)*	3 (9.68)	0 (0.00)	0.365	0.714
Cefpodoxime	5 (100.00)*	23 (95.83)	11 (78.57)	0.154	0.193	2 (50.00)*	12 (54.55)	12 (57.14)	1.000	1.000
Cefotaxime	80 (87.91)	24 (88.89)	10 (62.50) ^a^	0.041	0.148	20 (40.00)	13 (46.43)	12 (54.55)	0.512	0.791
Ceftazidime	82 (80.39)	25 (83.33)	11 (73.33)	0.678	0.678	5 (7.58)	4 (12.90)	7 (31.82) ^a^	0.024	0.281
Ceftizoxime	2 (50.00)*	15 (75.00)	7 (63.64)	0.663	0.678	2 (50.00)*	2 (10.00)	4 (40.00)	0.081	0.459
Imipenem	59(59.0%)	21(75.0%)	7(43.8%)	0.11	0.183	2 (3.17)	0 (0.00)	0 (0.00)	1.000	1.000
Colistin	0 (0.00)*	0 (0.00)	0 (0.00)	/	/	0 (0.00)*	1 (4.76)	0 (0.00)	0.511	0.791
Ampicillin-Sulbactam	83 (84.69)	8 (100.00)*	/	0.597	/	4 (6.35)	2 (22.22) *	/	0.160	0.680
Polymyxin B	0 (0.00)*	0 (0.00)*	/	/	/	/	0 (0.00)*	/	/	/
Ertapenem	5(71.4%)*	7(87.5%)*	/	0.44	/	0 (0.00)*	0 (0.00)*	/	/	/
Ceftriaxone	7 (77.78)*	8 (100.00)*	/	0.471	/	1 (16.67)*	3 (33.33)*	/	0.604	/

(1) Data are presented as number of resistant isolates/total number of isolates tested (resistance rate %). Denominators vary because some isolates were not tested for the corresponding antimicrobial agents.

(2) ^a^ Compared with the first period, ^b^ Compared with the third period, P<0.05.

(3) P values were derived from the chi-square test comparing resistance rates across the three periods. A P value of < 0.05 was considered statistically significant.

(4) P^#^ values were adjusted for multiple comparisons using the FDR procedure, applied separately to each antibiotic-pathogen combination group. An FDR-adjusted P^#^ < 0.05 was considered statistically significant.

(5) “/” indicates that the antibiotic is not applicable to the corresponding pathogen or that susceptibility testing was not performed.

(6) *: The number of tested isolates in this cell is < 10, and percentages should be interpreted with caution.

*K. pneumoniae*, *Klebsiella pneumoniae*; *E. coli*, *Escherichia coli.*

### Distribution and pathogen profile of fatal cases

Among the 894 neonates with confirmed sepsis, 19 died during hospitalization or on the day of treatment withdrawal due to severe complications, resulting in an in-hospital all-cause mortality rate of 2.13%. The mortality rate was higher in preterm neonates than in full-term neonates (3.83% vs. 1.20%, P = 0.009), whereas no statistically significant difference was observed between LOS and EOS (2.27% vs. 1.37%, P = 0.754). Stratified by study period, the in-hospital all-cause mortality rate increased from 1.39% in the pre-pandemic period to 3.29% in the pandemic period and 5.38% in the post-pandemic period. After stratification by gestational age, the mortality rates for both preterm and full-term neonates showed an upward trend numerically, but the differences across the three periods were not statistically significant (preterm: P = 0.092; full-term: P = 0.151) (**Table 7**).

**Table 7:** Comparison of mortality rates of neonatal sepsis among different populations and across different periods.

Factor	Total (N)	Deaths (n)	Mortality (%)	*P*
Overall	894	19	2.13	/
Gestational age				0.009
Preterm	313	12	3.83	
Full-term	581	7	1.20	
Age of onset				0.733
EOS	143	2	1.40	
LOS	751	17	2.26	
Study period				0.023
Pre-pandemic	649	9	1.39	
Pandemic	152	5	3.29	
Post-pandemic	93	5	5.38	
Preterm neonates				0.092
Pre-pandemic	211	5	2.40	
Pandemic	59	4	6.78	
Post-pandemic	43	3	6.98	
Term neonates				0.151
Pre-pandemic	438	4	0.91	
Pandemic	93	1	1.08	
Post-pandemic	50	2	4.00	

EOS, Early-onset sepsis; LOS, Late-onset sepsis.

**Figure 2:**
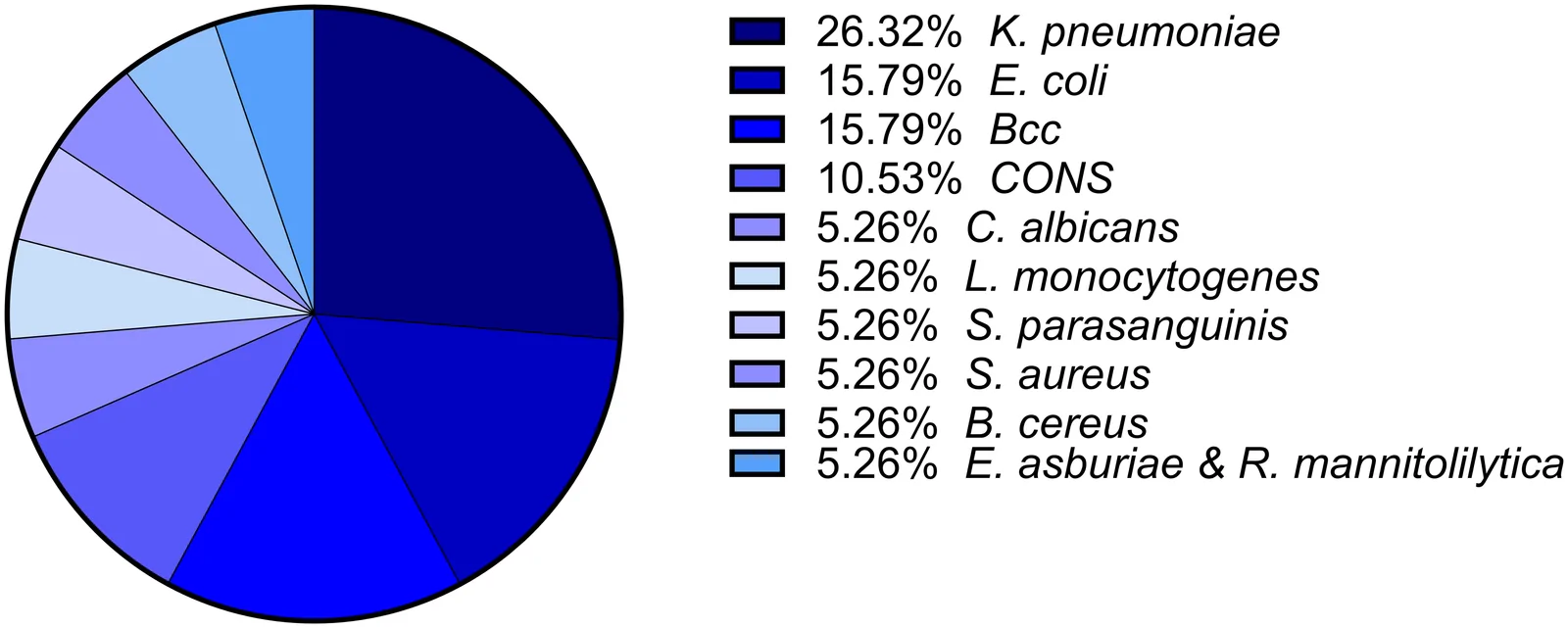
Distribution of pathogens among fatal neonatal cases.

Abbreviations: *K. pneumoniae, Klebsiella pneumoniae; E. coli, Escherichia coli; Bcc, Burkholderia cepacia complex;* CoNS, *Coagulase-negative staphylococci; C. albicans, Candida albicans; L. monocytogenes, Listeria monocytogenes; S. parasanguinis, Streptococcus parasanguinis; S. aureus, Staphylococcus aureus; B. cereus, Bacillus cereus; E. asburiae, Enterobacter asburiae; R. mannitolilytica, Ralstonia mannitolilytica.*

A total of 21 pathogens were isolated from the 19 fatal cases. Among these, *K. pneumoniae* was identified in 5 deaths (26.32%), *E. coli* and the *Burkholderia cepacia complex* (*Bcc*) in 3 deaths each (15.79%), CoNS in 2 deaths (10.53%), and *C. albicans*, *L. monocytogenes*, *Streptococcus parasanguinis* (*S. parasanguinis*), *S. aureus*, and *Bacillus cereus* (*B. cereus*) in 1 death each (5.26%). One death (5.26%) involved polymicrobial infection with *Enterobacter asburiae* (*E. asburiae*) and *Ralstonia mannitolilytica* (*R. mannitolilytica*) (**Figure 2**).

## Discussion

Neonatal sepsis is a leading cause of mortality and long-term disability among newborns worldwide (Fleischmann-Struzek et al., 2018). Its pathogen spectrum and antimicrobial resistance are influenced by regional factors, healthcare standards, and infection control measures, leading to substantial spatiotemporal heterogeneity (Li et al., 2023). The COVID-19 pandemic profoundly altered the epidemiological profile of many infectious diseases, while widespread prevention measures effectively curbed SARS-CoV-2 transmission (Chow et al., 2023; Zhang et al., 2023). However, no long-term study covering the full pandemic cycle, from pre-pandemic to strict containment and post-control periods, has been conducted on neonatal sepsis in a populous province of central China. This leaves a gap in evidence for adjusting post-pandemic infection control strategies. To address this gap, the present study analyzed 10 consecutive years of surveillance data from Henan Children’s Hospital, including 894 confirmed neonatal sepsis cases. For the first time, it revealed the dynamic changes in pathogen spectrum, antimicrobial resistance, and clinical outcomes of neonatal sepsis across different COVID-19 control phases. These findings provide locally derived, high-quality evidence to optimize empirical anti-infective strategies in the neonatal intensive care unit (NICU) for this region and other areas with similar demographic characteristics.

Following the implementation of COVID-19 prevention and control policies, the average annual number of admissions to the neonatal department of the hospital decreased from 4,134 before the pandemic to 2,473 during the pandemic, then slightly rebounded to 2,530 in the post-pandemic period, without returning to the pre-pandemic level. This marked reduction in total admissions was closely associated with the widespread prevention measures. Enhanced hand hygiene, social distancing, and visitor restrictions effectively reduced pathogen transmission in community settings (Talic et al., 2021). Meanwhile, traffic controls and parental avoidance of non-essential medical visits lowered the hospitalization demand for children with mild common infections (Anderson et al., 2020). These trends are consistent with the general decline in pediatric hospitalizations observed in many regions worldwide during the pandemic (Kruizinga et al., 2021; Bozzola et al., 2022). During the same period, the incidence of neonatal sepsis decreased from 3.14% to 2.05%, and further dropped to 1.84% in the post-pandemic period. Notably, the reduction in the average annual number of confirmed cases far exceeded that in total admissions. Despite a slight rebound in admissions in the post-pandemic period, the incidence continued to decline. A year by year decomposition analysis of data from the post-pandemic period revealed no statistically significant differences between 2023 and 2024 in sepsis incidence, all-cause mortality rate, CoNS detection rate, or the proportion of preterm neonates. These findings further support the robustness of the trends observed during this period. These findings suggest that the reduction in sepsis burden was not simply due to a shrinking patient volume, but rather reflects a genuine and sustained protective effect of pandemic-related prevention measures on infection control both in hospital and in the community.

The protective effect of pandemic measures was further contextualized by the baseline characteristics of the study population. Baseline characteristics across the three pandemic phases were well comparable. No significant differences were observed in sex distribution, mode of delivery, type of sepsis, or age at onset across the phases, supporting the reliability of the study findings. LOS accounted for 84.0% of cases and EOS for 16.0%, and this distribution pattern remained stable across all pandemic phases, indicating that the pandemic and its associated prevention measures did not alter the compositional pattern of EOS versus LOS. This finding is consistent with multi-center epidemiological data from both domestic and international sources (Giannoni et al., 2018; Al-Shehab et al., 2025; Yu et al., 2026). Of note, the proportion of preterm neonates increased from 32.5% before the pandemic to 46.2% after the pandemic, while median gestational age and birth weight showed a progressive decline. Several factors may explain this change. First, advances in perinatal medicine have enabled the survival of more extremely preterm neonates (Getaneh et al., 2025). Second, the impact of the pandemic on the accessibility of antenatal care may have indirectly increased the risk of preterm birth (Asumadu et al., 2024; Opoku-Boateng et al., 2024; Silverstein et al., 2025). Third, the reduced hospitalization demand for children with common infections during the pandemic concentrated the NICU admission profile toward critically ill neonates such as preterm and low-birth-weight neonates.

The distribution of culture types among the 894 confirmed neonates was analyzed. Overall, 89.93% had positive blood cultures only, 6.04% had positive CSF cultures only, and 4.03% had both blood and CSF cultures positive, indicating that approximately 10% of the neonates had concurrent purulent meningitis. This proportion is consistent with domestic and international reports on the prevalence of meningitis in neonatal sepsis (Giannoni et al., 2018; Li et al., 2025). Notably, 6.0% of the neonates were diagnosed solely by CSF culture, suggesting that reliance on blood culture alone may miss cases of isolated central nervous system infection (Aleem and Greenberg, 2019). These findings support the clinical value of routine CSF examination in neonates with suspected sepsis, particularly for those with neurological abnormalities or confirmed bloodstream pathogens, in defining the extent of infection, guiding antibiotic selection, and determining prognosis (Wiswell et al., 1995).

Stratified annual analysis further revealed long-term shifts in the pathogen spectrum. Between 2015 and 2024, Gram-positive bacteria detection decreased from 61.7% to 28.3%, while Gram-negative bacteria increased from 34.0% to 61.7%. Fungal rates fluctuated without clear trend. Across the three periods, CoNS detection decreased progressively from 36.6% to 9.8%, remaining significant after FDR adjustment in the overall study population, full-term neonates, and LOS subgroups. By contrast, although a downward trend was observed numerically in preterm neonates and EOS subgroups, it did not reach statistical significance after adjustment. By contrast, although a downward trend was observed numerically in preterm neonates and EOS subgroups, it did not reach statistical significance after adjustment. The detection rate of *E. coli* increased and becoming the leading pathogen in full-term neonates and LOS cases in the post-pandemic period. This restructuring may partially reflect differential impacts of prevention measures. As skin-colonizing bacteria transmitted via contact, CoNS may be effectively controlled by enhanced hand hygiene and contact isolation (Li et al., 2023). Conversely, Gram-negative bacilli from intestinal or environmental sources have more complex transmission routes, which may limit the impact of hand hygiene alone (Barcellini et al., 2025). Notably, CoNS detection did not rebound after measures were lifted, suggesting some enhanced practices became routine. Meanwhile, the post-pandemic rise in Gram-negative bacteria may relate to resumed social activities, increased environmental exposure, and altered antibiotic use patterns. However, that this study did not directly measure process indicators such as hand hygiene compliance or environmental cleanliness; these potential associations should therefore be interpreted with appropriate caution and explored in future studies.

Along with pathogen spectrum shifts, *L. monocytogenes* deserves attention for causing severe EOS via vertical transmission, making it a key target for perinatal infection control (Koopmans et al., 2023). Its detection rate was higher in the post-pandemic period (5.9%) than before (1.3%) or during (3.0%) the pandemic. Among EOS cases, its proportion rose from 8.3% to 26.3%, and after FDR adjustment, this trend remained statistically significant in the overall study population but lost significance in the EOS subgroup. This loss of significance may be partly attributable to the limited sample size of this subgroup, as only 19 EOS cases were documented during the post-pandemic period, which substantially reduced the statistical power for detecting period-specific differences. Several mechanisms may explain this rise. Standardized screening and prevention for Group B Streptococcus have controlled traditional EOS pathogens, relatively highlighting *L. monocytogenes*. Maternal dietary shifts during the pandemic, possibly increased consumption of ready-to-eat foods, and post-pandemic resumption of social activities with greater food circulation may have expanded exposure risks. Given the high disability and mortality of *L. monocytogenes* infection, it should be routinely considered in the differential diagnosis of EOS, and empirical anti-infective regimens covering this pathogen should be initiated promptly.

In addition to *L. monocytogenes*, the detection rate of *P. aeruginosa* showed a continuous upward trend, increasing from 0.3% in the pre-pandemic to 1.2% during the pandemic and 5.9% in the post-pandemic. This trend remained statistically significant after FDR adjustment in the overall study population. In the preterm neonate subgroup, however, the differences were no longer significant after adjustment, and no independent upward trend was confirmed. *P. aeruginosa* is a typical opportunistic pathogen causing NICU nosocomial infections via contaminated medical devices or healthcare workers’ hands (Letizia et al., 2025). Its persistent rise suggests weaknesses in infection control within the unit. During the pandemic, resources were preferentially allocated to respiratory protection, while environmental disinfection and medical device cleaning for contact transmission received relatively less attention (Weimer et al., 2022). This reallocation may have increased nosocomial transmission risk. However, these explanations are indirect; the increased detection rate could also relate to factors such as more frequent testing or concentrated admission of critically ill neonates. Therefore, in the post-pandemic era, infection control measures targeting contact transmission and waterborne environmental pathogens need strengthening, including environmental disinfection, invasive procedure management, and contact isolation.

Pathogen spectrum patterns differed markedly between preterm and full-term neonates. Among preterm neonates, Gram-negative bacteria predominated across all three periods, with *K. pneumoniae* consistently the leading pathogen, indicating a relatively stable structure consistent with Ni et al (Ni et al., 2025). After FDR adjustment, the changes in detection rates for all specific bacterial species within the preterm neonate subgroup no longer reached statistical significance. This finding suggests that, after controlling for multiple comparisons, the available data are insufficient to confirm genuine temporal variations in pathogen distribution within this subgroup. This lack of significance may be partly attributable to the relatively small sample size of the preterm neonate subgroup (n = 334), which may have limited the statistical power to detect more modest period-related differences. In contrast, full-term neonates exhibited a marked shift from Gram-positive predominance before the pandemic to Gram-negative predominance in the post-pandemic period. This change remained statistically significant after FDR adjustment. This differential pattern likely reflects differences in immune maturity, duration of hospital stay, and intensity of medical exposure between the two populations. Regarding sepsis type, Gram-positive bacteria predominated in EOS cases. However, the shift in the predominant pathogen from CoNS to *L. monocytogenes* was not significant after FDR adjustment, likely due to the limited number of EOS cases (only 19 cases in the post-pandemic period), which reduced the statistical power to detect period-related changes. LOS underwent a transition from Gram-positive to Gram-negative predominance, a pattern highly consistent with that seen in full-term neonates. These differences mirror the distinct infection routes: EOS primarily associated with vertical transmission, and LOS with horizontal transmission within the hospital (Strunk et al., 2024). The rising proportion of *L. monocytogenes* in EOS underscores the urgent need to strengthen prevention of foodborne infections during pregnancy. The persistently increasing proportion of *E. coli* in LOS imposes higher demands on infection control practices in the NICU.

Among Gram-positive bacteria, CoNS remained highly susceptible to vancomycin and linezolid, consistent with multiple surveillance studies, supporting these agents as core options for drug-resistant CoNS infections (Mohan et al., 2002; Li et al., 2022; Song et al., 2022). The resistance rate of CoNS to clindamycin showed a downward numerical trend, although this change did not reach statistical significance after FDR adjustment. Whether this trend holds clinical relevance requires further investigation. *E. faecium* Resistance rates of CoNS to ciprofloxacin and levofloxacin showed an upward trend, potentially related to inappropriate fluoroquinolone use. Although Chinese guidelines do not recommend routine fluoroquinolone use in neonates, salvage therapy for multidrug-resistant infections still exerts selective pressure (Subspecialty Group of Neonatology tSoP et al., 2024). The resistance rate to ciprofloxacin decreased from 88.5% before the pandemic to 25.0% after the pandemic, and to tetracycline from 76.0% to 50.0%, suggesting reduced selective pressure from these agents. The resistance rate to erythromycin, after a transient decline during the pandemic, rebounded to 100% in the post-pandemic period, a fluctuation possibly related to adjustments in antimicrobial use strategies during the pandemic. *E. faecium* remained 100% susceptible to linezolid, vancomycin, and tigecycline, providing reliable therapeutic options for multidrug-resistant enterococcal infections. However, its resistance rates to penicillins and macrolides remained persistently high, indicating that these agents may not be suitable for empirical therapy of *E. faecium* sepsis.

Among Gram-negative bacteria, *K. pneumoniae* maintained a high resistance rate to third-generation cephalosporins throughout the study period, indicating these agents are no longer appropriate as first-line empirical therapy (Kakaraskoska Boceska et al., 2024; Ni et al., 2025; Sau et al., 2025). Its resistance to carbapenems fluctuated, increasing during the pandemic and declining afterward, possibly due to higher carbapenem use from severe infections during the pandemic and strengthened infection control curbing nosocomial transmission after the pandemic (AlBahrani et al., 2023). Global meta-analyses report a pooled carbapenem-resistant *K. pneumoniae* (CRKP) prevalence of only 0.3% in neonates (Hu et al., 2023). Asian multi-center studies show rates of 16.3% in Chinese preterm neonates and 17.1% in the NeoSEAP study (Dickson et al., 2025; Yu et al., 2026). In contrast, the resistance rate in the present study ranged from 42.9% to 87.5%, notably higher, potentially due to selection bias from referral cases, cumulative carbapenem use, and possible short-term CRKP clone outbreaks (Hu et al., 2023). In contrast to *K. pneumoniae*, *E. coli* maintained relatively low resistance rates to beta-lactamase inhibitor combinations, Amikacin, Carbapenems, and Polypeptides, preserving reliable empirical options. However, the resistance rate to cephalosporins increased numerically from 7.6% to 31.8%, but this change did not reach statistical significance after FDR adjustment. Notably, the post-pandemic resistance rate of 31.8% remains relatively high, suggesting that its clinical relevance merits further attention. indicating persistent selective pressure from third-generation cephalosporins and underscoring the need for rigorous evaluation before empirical use. Resistance rates to cefuroxime and fluoroquinolones remained high throughout the study period, further limiting their empirical value in neonatal sepsis.

Along with the evolving resistance patterns, the in-hospital all-cause mortality rate increased progressively across the three phases, from 1.4% before the pandemic to 3.3% during the pandemic and 5.4% after the pandemic, P = 0.023. The overall mortality rate in this center (2.1%) was lower than the global average of 11%–19% reported in low-and middle-income countries (Fleischmann-Struzek et al., 2018), possibly due to selection factors: as a regional referral center, the hospital primarily admits neonates referred from community settings or other hospitals, excluding those too critically ill to tolerate transport from their birth hospital, and some neonates with poor prognoses were not admitted or were discharged against medical advice and died thereafter, deaths not captured in our statistics. The rise in overall mortality during the post-pandemic period may reflect multi-level indirect associations with COVID-19. First, maternal SARS-CoV-2 infection has been linked to preterm birth, fetal distress, and adverse neonatal outcomes (McClymont et al., 2022). Second, the prolonged non-pharmacological interventions implemented during the strict pandemic control period reduced population exposure to common pathogens, which may have affected the immune status of women of childbearing age to some extent (Cohen et al., 2021). After restrictions were lifted, concentrated exposure to SARS-CoV-2 and other respiratory pathogens may have further increased the risk of adverse neonatal outcomes (Gurol-Urganci et al., 2021). Third, in the post-pandemic period, rebounds in respiratory syncytial virus and influenza virus infections may have contributed to disease severity and mortality in neonates (Li et al., 2023a; Li et al., 2023). These factors, together with rising preterm births and evolving antimicrobial resistance, may have collectively shaped the multifactorial context for increased neonatal sepsis mortality (Marsh et al., 2023). Given the limited number of deaths (n = 19), these interpretations remain tentative and warrant validation in larger multicenter studies.

Analysis of fatal cases further supported these drivers. Among the 19 neonates who died, 21 pathogens were isolated, with Gram-negative bacteria predominating (14 isolates, 66.7%). *K. pneumoniae* ranked first (5 cases, 26.3%), followed by *E. coli* and *B. cepacia* complex (3 cases each, 15.8%), confirming Gram-negative bacteria as both the main driver of pathogen spectrum shift and the core pathogens linked to poor prognosis. Several rare but highly lethal pathogens also appeared, including *B. cepacia* complex, *C. albicans*, *L. monocytogenes*, and B*. cereus*, suggesting empirical therapy should cover such pathogens. *C. albicans* was the only fungus detected, and the affected neonate was preterm, a group at high risk for fungal infections (Weimer et al., 2022). However, systematic antifungal susceptibility testing was not performed in this study, so resistance characteristics could not be assessed. Clinicians should adjust empirical anti-infective regimens based on dynamic changes in pathogen spectrum and resistance, and select empirical antifungal regimens based on local resistance surveillance data, to avoid treatment failure and curb worsening prognosis.

This study has the following strengths: (1) With a study span of up to 10 years, it fully covered the three periods before, during, and after the COVID-19 pandemic, this design enabled a systematic evaluation of both the short-term and long-term effects of pandemic-related prevention measures on the pathogen spectrum of neonatal sepsis. (2) Stratified analyses by gestational age (preterm/full-term neonates) and age of onset (EOS/LOS) were performed simultaneously, revealing significant heterogeneity in pathogen spectrum changes across different populations. (3) The long-term consecutive observation systematically revealed a fundamental reshaping of the neonatal sepsis pathogen spectrum from Gram-positive predominance to Gram-negative predominance, and confirmed the sustained decline of CoNS detection rate without rebound. (4) For analyses of pathogen spectrum and resistance trends across the three periods, we used the Benjamini–Hochberg method to control the false discovery rate (FDR) for multiple comparisons. All core conclusions remained robust after FDR adjustment, enhancing the statistical reliability of our findings. (5) It identified the dual characteristics of the current resistance landscape, in which some agents have become highly resistant while key agents retain good susceptibility, thereby guiding the optimization of empirical treatment strategies.

However, this study has several limitations. (1) The single-center retrospective design may introduce selection bias, and the generalizability of the findings awaits validation by multicenter prospective studies. (2) Only culture-positive cases were included; culture-negative neonates with clinical sepsis were not enrolled, which may lead to underestimation of the disease burden. (3) Due to the limited number of fungal isolates (67 strains), systematic antifungal susceptibility testing was not performed, and an in-depth discussion of fungal clinical characteristics and resistance trends was not conducted. Therefore, the pathogen spectrum changes revealed in this study primarily apply to bacterial pathogens. (4) Inherent limitations exist regarding the staging of the COVID-19 pandemic periods and causal inference. The present study used the implementation and adjustment of China’s pandemic prevention and control policies as the basis for period stratification. While this approach allowed observation of stage-specific changes in pathogen spectrum and antimicrobial resistance, multiple intertwined factors may have contributed to the observed trends, including alterations in healthcare-seeking behaviors, changes in antibiotic utilization patterns, advances in perinatal medicine, and shifts in medical care practices during the pandemic. Consequently, it is difficult to attribute the observed changes to any single measure or factor. Furthermore, the three study periods had varying durations (5 years pre-pandemic, 3 years during the pandemic, and 2 years post-pandemic period). Given its shorter length, the post-pandemic period was more susceptible to interference from short-term fluctuations or small-sample random errors. To mitigate the bias arising from these unequal time periods, we used annualized data in intergroup comparisons of hospitalization volumes and incidence rates, and performed a year by year decomposition analysis of the core indicators during the post-pandemic period. The results showed no statistically significant differences in the core indicators between the two years of the post-pandemic period, suggesting that the aggregate findings from this period were not driven by abnormal fluctuations in any single year. These analyses support the reliability of the overall conclusions. The pathogen spectrum restructuring and resistance evolution revealed in this study should be interpreted cautiously within this complex context. (5) Susceptibility testing was limited to clinically submitted isolates, and non-submitted isolates or incomplete testing panels did not yield data. Instruments remained unchanged, but antimicrobial panels varied over the decade with evolving clinical practice. As a result, susceptibility data for some drugs were available only for specific periods, and sample sizes for certain drug-period combinations were small. Accordingly, resistance trend analyses, particularly when isolate numbers were limited in a given period, should be viewed as descriptive and warrant validation in larger prospective studies. Thus, resistance rate estimates based on small sample sizes should be interpreted with caution.

## Conclusion

Based on a single-center, 10-year dataset, this study reveals a fundamental reshaping of the neonatal sepsis pathogen spectrum during the COVID-19 pandemic: a shift from Gram-positive to Gram-negative predominance, with a sustained decline in CoNS that did not rebound in the post-pandemic period, and a concurrent rise in Gram-negative bacteria including *E. coli*. Regarding antimicrobial resistance, *K. pneumoniae* retained high resistance to third-generation cephalosporins, rendering these agents unsuitable for empirical therapy. In contrast, CoNS and *E. faecium* remain highly susceptible to vancomycin and linezolid; *E. coli* to carbapenems; and *K. pneumoniae* to carbapenems and tigecycline. These agents constitute the core empirical armamentarium. Importantly, Enhanced hand hygiene and contact isolation are consistent with a persistent decline in CoNS, a pathogen primarily transmitted via healthcare-associated contact, suggesting that such basic measures should be incorporated into routine NICU management.

## Data Availability

The data analyzed in this study is subject to the following licenses/restrictions: Due to ethical constraints, the data cannot be made freely available in the article or a public repository, as the study involved human participants. The corresponding author can provide the data used to support the study’s findings upon request. Requests to access these datasets should be directed to Tiewei Li (litieweind@163.com).
