# Supplementary material for: Trends in pathogens and antimicrobial resistance in culture-proven neonatal sepsis over a decade (2015–2024): the impact of COVID-19 pandemic
Source: Front Cell Infect Microbiol. 2026 Sep 3;16:1913607. doi: 10.3389/fcimb.2026.1913607 (PMC13581538; doi:10.3389/fcimb.2026.1913607)
Supplement: Supplementary file 1 [file Supplementaryfile1.docx]

**Supplementary**

Supplementary Table S1. Antimicrobial susceptibility of *K. pneumoniae* and *E. coli* to other agents across different periods.

| Antibiotics | *K. pneumoniae* | | | | | *E. coli* | | | | |
| --- | --- | --- | --- | --- | --- | --- | --- | --- | --- | --- |
|  | Pre-pandemic n, (%) | Pandemic  n, (%) | Post-pandemic n, (%) | *P* | *P^#^* | Pre-pandemic n, (%) | Pandemic  n, (%) | Post-pandemic n, (%) | *P* | *P^#^* |
| Aztreonam | 75 (73.53) | 23 (82.14) | 12 (75.00) | 0.683 | 0.683 | 12 (18.75) | 6 (20.00) | 7 (31.82) | 0.462 | 0.968 |
| Doxycycline | 3 (100.00)* | 12 (60.00) | 9 (56.25) | 0.487 | 0.576 | 1 (50.00)* | 3 (13.64) | 4 (18.18) | 0.484 | 0.968 |
| Trimethoprim-sulfamethoxazole | 35 (33.65) | 1 (3.57) ^ab^ | 5 (31.25) | 0.002 | 0.020 | 40 (62.50) | 23 (74.19) | 12 (54.55) | 0.314 | 0.968 |
| Ciprofloxacin | 52 (51.49) | 23 (85.19) ^ab^ | 8 (50.00) | 0.006 | 0.026 | 35 (57.38) | 19 (67.86) | 11 (50.00) | 0.428 | 0.968 |
| Minocycline | 2 (25.00)* | 12 (42.86) | 5 (31.25) | 0.575 | 0.623 | 1 (14.29)* | 1 (3.33) | 3 (13.64) | 0.293 | 0.968 |
| Moxifloxacin | 15 (53.57) | 25 (83.33) ^ab^ | 5 (33.33) | 0.003 | 0.020 | 10 (66.67) | 17 (60.71) | 11 (50.00) | 0.570 | 0.998 |
| Nalidixic acid | 4 (80.00)* | 21 (87.50) | 1 (100.00)* | 0.612 | / | 4 (100.00)* | 20 (95.24) | 10 (100.00) | 1.000 | 1.000 |
| Tetracycline | 49 (47.12) | 18 (60.00) | 7 (46.67) | 0.448 | 0.576 | 46 (70.77) | 20 (68.97) | 14 (63.64) | 0.823 | 1.000 |
| Cefepime | 85 (83.33) | 24 (82.76) | 11 (68.75) | 0.355 | 0.513 | 19 (30.16) | 8 (25.81) | 6 (27.27) | 0.900 | 1.000 |
| Cefuroxime | 13 (86.67) | 50 (92.59) ^b^ | 22 (73.33) | 0.045 | 0.098 | 5(35.71) | 28(52.83) | 25(58.14) | 0.345 | 0.968 |
| Cephalothin | 1 (100.00)* | 20 (95.24) | 9 (69.23) | 0.089 | 0.165 | 2 (66.67)* | 8 (57.14) | 8 (57.14) | 1.000 | 1.000 |
| Cefotetan | 2 (50.00)* | 17 (70.83) ^b^ | 3 (23.08) | 0.011 | 0.029 | 0 (0.00)* | 1 (4.35) | 0 (0.00) | 1.000 | 1.000 |
| Cefazolin | 88 (98.88) | 8 (100.00)* | 3 (100.00)* | 1.000 | / | 28 (87.50) | 3 (60.00)* | 2 (100.00)* | 0.290 | / |
| Tobramycin | 5 (41.67) | 13 (46.43) | 3 (20.00) | 0.256 | 0.416 | 0 (0.00)* | 3 (9.68) | 0 (0.00) | 0.365 | 0.968 |
| Levofloxacin | 49 (48.04) | 22 (81.48) ^a^ | 8 (50.00) | 0.008 | 0.026 | 32 (54.24) | 17 (58.62) | 11 (50.00) | 0.827 | 1.000 |
| Nitrofurantoin | 6 (75.00)* | 5 (100.00)* | / | 0.487 | / | 0 (0.00)* | 0 (0.00)* | / | / | / |
| Chloramphenicol | 35 (34.31) | 2 (25.00)* | / | 0.715 | / | 5 (7.69) | 2 (25.00)* | / | 0.167 | / |
| Gentamicin | 62 (59.05) | 5 (62.50)* | / | 1.000 | / | 30 (46.15) | 6 (66.67)* | / | 0.302 | / |
| Cefoxitin | 3 (50.00)* | 7 (87.50)* | / | 0.245 | / | 0 (0.00)* | 2 (25.00)* | / | 0.473 | / |
| Fosfomycin | / | / | / | / | / | 0 (0.00)* | 0 (0.00)* | / | / | / |

Notes: (1) Data are presented as number of resistant isolates / total number of isolates tested (resistance rate %). Denominators vary because some isolates were not tested for the corresponding antimicrobial agents.

(2) P values were derived from the chi‑square test comparing resistance rates across the three periods. A P value of < 0.05 was considered statistically significant.
(3) ^a^ compared with the first period; ^b^ compared with the third period, P<0.05.
(4) P^#^ values were adjusted for multiple comparisons using the Benjamini‑Hochberg false discovery rate (FDR) procedure, applied separately to each antibiotic‑pathogen combination group. An FDR‑adjusted P^#^ < 0.05 was considered statistically significant.
(5) “/” indicates that the antibiotic is not applicable to the corresponding pathogen or that susceptibility testing was not performed.
(6) *: The number of tested isolates in this cell is < 10, and percentages should be interpreted with caution.

Abbreviations: *K. pneumoniae*, *Klebsiella pneumoniae*; *E. coli*, *Escherichia coli.*

Supplementary Table S2. Year-by-Year Comparison of Core Indicators in the Post-Pandemic Period

| Indicator | 2023 | 2024 | *P* |
| --- | --- | --- | --- |
| Sepsis incidence (%) | 1.59 | 2.07 | 0.206 |
| All‑cause mortality rate (%) | 7.69 | 3.70 | 0.646 |
| CoNS detection rate (%) | 11.90 | 8.33 | 0.646 |
| Proportion of preterm neonates (%) | 41.03 | 59.26 | 0.083 |

Note: P values were derived from chi‑square tests or Fisher's exact tests, as appropriate. All tests were two‑sided, and P < 0.05 was considered statistically significant.

Abbreviations: CoNS, *coagulase-negative staphylococci.*

Supplementary Figure 1. Incidence rates of neonatal sepsis across the three study periods.

Note: The left y‑axis (bars) represents the average annual number of admissions, and the right y‑axis (line) represents the incidence rate of neonatal sepsis (%). Given that the three study periods had unequal durations (5 years pre‑pandemic, 3 years during the pandemic, and 2 years post‑pandemic period), all intergroup comparisons of hospital admission volumes were based on annualized data.

Supplementary Figure 2. Pathogen distribution and temporal trends (2015–2024).

(A) Distribution of positive cultures among the 894 confirmed neonatal sepsis cases.
(B) Annual detection rates of Gram‑positive bacteria, Gram‑negative bacteria, and fungi.

Abbreviation: CSF, cerebrospinal fluid.
